# Mechanisms of Nutritional Resource Exploitation by Insects

**DOI:** 10.3390/insects11090570

**Published:** 2020-08-25

**Authors:** Sara D. Leonhardt, Mathieu Lihoreau, Johannes Spaethe

**Affiliations:** 1 Plant-Insect-Interactions Group, Technical University of Munich, Hans-Carl-von-Carlowitz-Platz 2, 85354 Freising, Germany; 2 Research Center on Animal Cognition (CRCA), Center for Integrative Biology (CBI); CNRS, University Paul Sabatier - Toulouse III, 31062 Toulouse, France; mathieu.lihoreau@univ-tlse3.fr; 3 Department of Behavioral Physiology and Sociobiology, Biozentrum, University of Würzburg, Biozentrum, Am Hubland, 97074 Würzburg, Germany; johannes.spaethe@uni-wuerzburg.de

## Abstract

Insects have evolved an extraordinary range of nutritional adaptations to exploit other animals, plants, bacteria, fungi and soils as resources in terrestrial and aquatic environments. This special issue provides some new insights into the mechanisms underlying these adaptations. Contributions comprise lab and field studies investigating the chemical, physiological, cognitive and behavioral mechanisms that enable resource exploitation and nutrient intake regulation in insects. The collection of papers highlights the need for more studies on the comparative sensory ecology, underlying nutritional quality assessment, cue perception and decision making to fully understand how insects adjust resource selection and exploitation in response to environmental heterogeneity and variability.

## 1. Nutritional Resource Exploitation—An Ongoing Challenge in a Highly Variable World

Insects need to acquire specific amounts and ratios of nutrients to develop, survive and raise offspring [1]. Finding and accessing the right mixtures of nutrients in nature can be challenging, as food resources are often highly variable in their chemical/nutritional profile, and do not reliably provide the nutritional profile that best meets the insect’s nutritional needs [2,3]. Heterogeneity in resource quality is particularly striking for pollinating insects, such as bees, which must visit thousands of flowers to feed their progeny with nectar and pollen [4]. The study of Venjakob and colleagues [5] highlights a high degree of variation in the nutritional quality (i.e., carbohydrate and amino acid composition and ratio) of nectar among individual field scabious (*Knautia arvensis*) plants growing in the same and different plant communities. Russo and colleagues [6] show that elevated temperatures, as may be expected in the future as a consequence of global climate change, additionally affect variations in pollen chemistry. Specifically, lipid content increased in pollen of the thistle *Carduus nutans* when plants were grown at higher temperatures. Increased lipid contents strongly decrease pollen collection, survival and reproductive fitness in bumble bees (*Bombus terrestris*) [7], and may therefore reduce thistle pollen suitability and uptake by bees in warmer environments. This new insight into intra-specific variation in resource chemistry adds to the known inter-specific variation in resource chemistry observed for different plant species, which is also nicely illustrated by the study of Vaudo and colleagues [8] who document inter-specific variation in protein to lipid (P:L) ratios in the pollen of more than 80 plant species. Disentangling the causes underlying the observed variation in resource chemistry within and between plant species represents a challenging but intriguing scope for future research, as such variation can strongly affect resource suitability and attractiveness. In the case of flower visiting insects, this can directly influence pollination success, with consequences for plant-pollinator interaction networks in different environments.

## 2. Even More Complex? Linking Nutrition and Social Regulation in Eusocial Insects

In eusocial insects, such as ants and several bee species, the challenge of coping with variation in resource chemistry is further amplified by often largely different caste- or symbiont-specific nutritional needs among colony members, which all need to be met simultaneously, as discussed by Crumière and colleagues [9]. Moreover, social organization, which typically implies division of labor, appears to be closely linked to nutritional regulation. In most insects, dietary restriction, and in particular a reduced protein intake, increases lifespan [3]. Interestingly, in the eusocial honey bee (*Apis mellifera*), dietary restriction shortens lifespan in colony-living workers, because their maturation is accelerated, and they start foraging comparatively earlier when food deprived, as reviewed by Ihle and colleagues [10]. Similar effects can be observed when nutrient sensing insulin/insulin-like signaling (IIS) and target of rapamycin (TOR) pathways are suppressed [11]. As insulin signaling is involved in the division of labor, Ihle and colleagues [10] postulated a link between the regulation of nutritional intake and longevity on one hand and division of labor on the other hand. Using RNA interference, they downregulated the *insulin receptor substrate* (*irs*) gene in two honey bee lines. Treated bees were then brought back into their mother colonies, and the onset of foraging and lifespan were recorded. Individuals with suppressed *irs* showed an earlier foraging onset and did not live as long, suggesting that the IIS pathway links nutritional regulation with behavioral maturation and life span.

## 3. Physiological Adaptations to Cope with Chemically Defended, Highly Variable and Nutritionally Inappropriate Resources

Insects have evolved sophisticated traits and strategies to locate, memorize, select and exploit valuable nutritional resources, in complex and variable environments. The exploitation and use of pollen in the plant-pollinator mutualism are particularly interesting, because pollen has an intriguing dual role. It carries the male gametes and thus ensures plant fertilization, and it can (in some plant species) serve as a reward to pollinators, as reviewed by Ruedenauer and colleagues [12]. Many plant species therefore morphologically, mechanically and/or chemically protect pollen, to restrict its exploitation to the most effective pollinators [13]. Vanderplanck and colleagues [14] investigated how such restriction is achieved in *Taraxacum* (Asteraceae), which are known for their specialized pollinator interactions. The authors show that a *Taraxacum* diet strongly reduces offspring production and health in a generalist bumble bee (*B. terrestris*). This effect is unlikely to be caused by pollen mechanical properties (e.g., a thick pollen wall), but rather a consequence of lacking essential dietary components or of the presence of toxic compounds that interfere with physiological processes (i.e., chemical defense).

While most solitary bees and several primitively eusocial bumble bees provision their offspring with pollen and nectar without (chemically) modifying it, the eusocial stingless bees and honey bees convert the collected nectar into honey [15]. For honey production, workers add bee-secreted peptides and proteins as essential nutrients and as antimicrobials to prevent honey from molding. Lewkowski and colleagues [16] investigated whether the composition of peptides and proteins in the honey-like product produced by caged honey bee workers depended on the presence of proteins in the starting nectar-like solutions (honey, sucrose with varying additives of proteins and plant secondary metabolites, or pure sucrose). They found that, irrespective of the starting material, the composition of metabolism enzymes and royal jelly characteristic proteins in the final product was similar, suggesting that the workers secreted variable amounts of bee-specific protein into the honey. In doing so, bees seem to be able to adjust the nutritional quality of variable nectar-like solutions, differing in their composition, which likely optimizes the preservation and nutritional quality of this important food source.

## 4. The Role of Floral Diversity for Compensating Inter- and Intra-Specific Variation in Resource Chemistry and Availability

Insects that cannot alter the nutritional composition of allocated food resources may compensate for inter- and intra-specific variation in resource chemistry by relying on resource diversity. For example, floral diversity is known to ensure a continuous resource supply which allows for a continuously high food intake and an increase in the quantity of stored food resources [17]. Floral diversity can also increase the nutritional quality of food (e.g., through diluting toxic plant compounds [18,19]). However, high floral diversity does not automatically imply high food nutritional quality [17,20], as composition also matters. Diets with ideal nutrient composition may be most easily composed in habitats with high resource diversity, where animals can mix resources with variable nutrient contents obtained from different plant species to compose a high quality diet, as suggested by the study of Trinkl and colleagues [21]. The authors analyzed the nutritional composition of larval provisions obtained from stingless bee colonies located in various environments differing in floral diversity. They show that both the proportion of beneficial fatty acids and the P:L ratio increased with increasing plant species richness, in both larval provisions and the surrounding environment. These implications highlight the importance of biodiverse environments for the fitness and population growth of many herbivorous insect species, because they provide a continuous supply of resources. As insects provide a major food source for several higher trophic levels (e.g., birds), fitness benefits induced by resource diversity at lower trophic levels may well translate into increased population sizes at higher trophic levels.

## 5. Towards a More Integrative Research on Insect Nutrition

It has long been known that insects primarily choose their food based on qualitative nutrient contents. For example, Grund-Mueller and colleagues [22] show that adding protein or amino acids to a sucrose diet is not sufficient to support the longevity and reproduction of adult bumble bees (*B. terrestris*). Other nutrients (such as lipids and micro-nutrients) are required for physiological maintenance. Moreover, several studies indicate that species-specific target ratios of micro- and macro-nutrients are critical for the health and fitness of animals in general [3], and insects in particular [23,24]. However, for most insect species, we do not know their precise nutritional needs, level of tolerance towards deviations from an optimal nutrient intake, or how nutrient targets are affected by trophic interactions, social organization and environmental variation. Contributions in this special issue highlight the need for a better integration of mechanistic studies, with more classical descriptions of nutritional requirements, to fully understand the behavioral, physiological and/or sensory adaptations that enable resource selection and exploitation by insects. Such integration can only be performed by considering mechanisms at multiple levels of organization (e.g., individuals, societies, host-parasites, competitors and predators) and their interactions across levels (as discussed in [9] and other recent studies [25,26,27]). Morimoto and Lihoreau [28] and Crumière and colleagues [9] highlight the importance of further developing existing concepts of nutrition research, such as the Geometric Framework of Nutrition (GFN [3], see description by authors), which has proven to be an extremely useful tool for assessing the impact of nutrient ratios (e.g., P:L) in insects and animals in general. Morimoto and Lihoreau [28] call for open access GFN data as a basis to start developing comparative analyses, and they provide a template for structuring such data to facilitate meta-analyses using quantitative methods [28]. Studying mechanisms and comparing them across species will significantly leverage our understanding of nutritional resource exploitation by insects, which may prove to be precious information for long-term conservation plans.

## 6. An Open Question: Sensory Mechanisms—How to Make Sense of it All?

The observed variation in resource chemistry requires that insects consuming these resources are able to assess (e.g., taste) their chemical/nutritional profiles, in order to make appropriate foraging decisions. Numerous behavioral studies indicate that they are [29], but the underlying physiological and neuronal mechanisms are little understood, particularly with regard to the perception of macro-nutrients other than sugar (e.g., protein or fat). It has recently been shown that bumble bees (*B. terrestris*) can perceive all those amino acids that have a polarized functional group, in addition to the amino acid-specific amino- and carboxy-group [30]. Moreover, the bees do not differentiate between different amino acids, but between different concentrations of the same amino acid [30]. Interestingly, however, bumble bees did not differentiate between pure pollen and pollen enriched with amino acids [7], indicating that nutrients/compounds other than amino acids affect their pollen foraging decisions. In fact, when enriching pollen with fatty acids (instead of amino acids), bumble bees differentiated between pure and nutritionally enriched pollen [7]. This finding suggests that, although both amino acids and fatty acids are critical and can be perceived, the fatty acid cue is prioritized over the amino acid cue, when the bees receive both (nutritional) cues. The prioritization of fatty acids over amino acids is also in accordance with previous findings of Vaudo and colleagues [23], who showed that *B. impatiens* preferentially foraged on plants with pollen of high protein to fat content. The prioritized perception of a critical dietary component not only increases the chances of survival and thus reproduction, but also reduces the costs associated with cue perception. It may represent a sophisticated strategy of generalist consumers to efficiently exploit diverse resources through rapid quality assessment at low cost. This would allow them to maximally benefit from resource diversity, through selecting the most beneficial resources or resource mixture.

## 7. Conclusions

The studies published in this Special Issue highlight the need for more detailed and comparative studies on the sensory ecology of insects to investigate the factors (e.g., dietary specialization, the level of sociality [9] or the ability to modify resources (see e.g., [16]), determining nutritional quality assessment, cue perception and decision making across species to fully understand how insects adjust resource selection and exploitation in response to environmental heterogeneity and variability.

## References

[1] Simpson S.J., Clissold F.J., Lihoreau M., Ponton F., Wilder S.M., Raubenheimer D. (2015). Recent advances in the integrative nutrition of arthropods. Annu. Rev. Entomol..

[2] Jarau S., Hrncir M. (2009). Food Exploitation by Social Insects: Ecological, Behavioral and Theoretical Approaches.

[3] Simpson S.J., Raubenheimer D. (2012). The Nature of Nutrition: A Unifying Framework from Animal Adaptation to Human Obesity.

[4] von Frisch K. (1967). The Dance Language and Orientation of Bees.

[5] Venjakob C., Leonhardt S., Klein A.-M. (2020). Inter-individual nectar chemistry changes of field scabious, knautia arvensis. Insects.

[6] Russo L., Keller J., Vaudo A.D., Grozinger C.M., Shea K. (2020). Warming increases pollen lipid concentration in an invasive thistle, with minor effects on the associated floral-visitor community. Insects.

[7] Ruedenauer F.A., Raubenheimer D., Kessner-Beierlein D., Grund-Mueller N., Noack L., Spaethe J., Leonhardt S.D. (2020). Best be(e) on low fat: Linking nutrient perception, regulation and fitness. Ecol. Lett..

[8] Vaudo A.D., Tooker J.F., Patch H.M., Biddinger D.J., Coccia M., Crone M.K., Fiely M., Francis J.S., Hines H.M., Hodges M. (2020). Pollen protein:Lipid macronutrient ratios may guide broad patterns of bee species floral preferences. Insects.

[9] Crumière A.J.J., Stephenson C.J., Nagel M., Shik J.Z. (2020). Using nutritional geometry to explore how social insects navigate nutritional landscapes. Insects.

[10] Ihle K.E., Mutti N.S., Kaftanoglu O., Amdam G.V. (2019). Insulin receptor substrate gene knockdown accelerates behavioural maturation and shortens lifespan in honeybee workers. Insects.

[11] Clancy D.J., Gems D., Harshman L.G., Oldham S., Stocker H., Hafen E., Leevers S.J., Partridge L. (2001). Extension of life-span by loss of chico, a *Drosophila* insulin receptor substrate protein. Science.

[12] Ruedenauer F.A., Spaethe J., van der Kooi C.J., Leonhardt S.D. (2019). Pollinator or pedigree: Which factors determine the evolution of pollen nutrients?. Oecologia.

[13] Waser N.M., Ollerton J. (2006). Plant-Pollinator Interactions: From Specialization to Generalization.

[14] Vanderplanck M., Gilles H., Nonclercq D., Duez P., Gerbaux P. (2020). Asteraceae paradox: Chemical and mechanical protection of *Taraxacum* pollen. Insects.

[15] Wright G.A., Nicolson S.W., Shafir S. (2017). Nutritional physiology and ecology of honey bees. Annu. Rev. Entomol..

[16] Lewkowski O., Mureșan C.I., Dobritzsch D., Fuszard M., Erler S. (2019). The effect of diet on the composition and stability of proteins secreted by honey bees in honey. Insects.

[17] Kaluza B.F., Wallace H.M., Heard T.A., Minden V., Klein A.M., Leonhardt S.D. (2018). Social bees are fitter in more biodiverse environments. Sci. Rep..

[18] Irwin R.E., Cook D., Richardson L.L., Manson J.S., Gardner D.R. (2014). Secondary compounds in floral rewards of toxic rangeland plants: Impacts on pollinators. J. Agric. Food Chem..

[19] Eckhardt M., Haider M., Dorn S., Müller A. (2014). Pollen mixing in pollen generalist solitary bees: A possible strategy to complement or mitigate unfavourable pollen properties?. J. Anim. Ecol..

[20] Kaluza B.F., Wallace H.M., Keller A., Heard T.A., Jeffers B., Drescher N., Blüthgen N., Leonhardt S.D. (2017). Generalist social bees maximize diversity intake in plant species-rich and resource-abundant environments. Ecosphere.

[21] Trinkl M., Kaluza B.F., Wallace H.M., Heard T., Keller A., Leonhardt S.D. (2020). Floral species richness correlates with changes in the nutritional quality of larval diets in a stingless bee. Insects.

[22] Grund-Mueller N., Ruedenauer F.A., Spaethe J., Leonhardt S.D. (2020). Adding amino acids to a sucrose diet is not sufficient to support longevity of adult bumble bees. Insects.

[23] Vaudo A.D., Patch H.M., Mortensen D.A., Tooker J.F., Grozinger C.M. (2016). Macronutrient ratios in pollen shape bumble bee (*Bombus impatiens*) foraging strategies and floral preferences. Proc. Natl. Acad. Sci. USA.

[24] Filipiak M., Kuszewska K., Asselman M., Denisow B., Stawiarz E., Woyciechowski M., Weiner J. (2017). Ecological stoichiometry of the honeybee: Pollen diversity and adequate species composition are needed to mitigate limitations imposed on the growth and development of bees by pollen quality. PLoS ONE.

[25] Lihoreau M., Buhl J., Charleston M.A., Sword G.A., Raubenheimer D., Simpson S.J. (2015). Nutritional ecology beyond the individual: A conceptual framework for integrating nutrition and social interactions. Ecol. Lett..

[26] Pasquaretta C., Gómez-Moracho T., Heeb P., Lihoreau M. (2018). Exploring interactions between the gut microbiota and social behavior through nutrition. Genes.

[27] Simpson S.J., Raubenheimer D., Charleston M.A., Clissold F.J. (2010). Modelling nutritional interactions: From individuals to communities. Trends Ecol. Evol..

[28] Morimoto J., Lihoreau M. (2020). Open data for open questions in comparative nutrition. Insects.

[29] Nicholls E., Hempel de Ibarra N. (2017). Assessment of pollen rewards by foraging bees. Funct. Ecol..

[30] Ruedenauer F.A., Leonhardt S.D., Lunau K., Spaethe J. (2019). Bumblebees are able to perceive amino acids via chemotactile antennal stimulation. J. Comp. Physiol. A.

